# Bowel injury following gynecological laparoscopic surgery

**DOI:** 10.4314/ahs.v17i4.35

**Published:** 2017-12

**Authors:** Hassan M Elbiss, Fikri M Abu-Zidan

**Affiliations:** 1 Department of Obstetrics and Gynaecology, College of Medicine and Health Sciences, UAE University, 17666 Al-Ain, United Arab Emirates. helbiss@uaeu.ac.ae; 2 Department of Surgery, College of Medicine and Health Sciences, UAE University,17666 Al-Ain, United Arab Emirates. fabuzidan@uaeu.ac.ae

**Keywords:** Laparoscopy, gynaecology, injury, bowel, prevention, treatment

## Abstract

**Background:**

Bowel injury remains a serious complication of gynecological laparoscopic surgery. We aimed to review the literature on this topic, combined with personal experiences, so as to give recommendations on how to avoid and manage this complication.

**Methods:**

We performed a narrative review on bowel injury following gynecological laparoscopic surgery using PubMed covering prevention, diagnosis, and management. Search terms used were laparoscopy, gynaecology, injury, bowel, prevention, treatment.

**Results:**

Important principles of prevention include proper pre-operative evaluation and increased laparoscopic surgical skills and knowledge. High clinical suspicion is crucial for early diagnosis. Diagnostic workup of suspected cases includes serial abdominal examination, measuring inflammatory markers, and performing imaging studies including abdominal ultrasound and CT scan. When bowel injury is recognized during the first laparoscopic procedure then laparoscopic primary suturing could be tried although laparotomy may be needed. When diagnosis is delayed, then laparotomy is the treatment of choice. The role of robotic surgery and three-dimensional laparoscopic gynecological surgery on bowel injury needs to be further assessed.

**Conclusion:**

Early recognition of bowel injury is crucial for a favorable clinical outcome. A combined collaboration between gynecologists and general surgeons is important for timely and proper decisions to be made.

## Introduction

Laparoscopy has many advantages over open surgery including less post-operative pain, earlier return of normal bowel function, shorter hospital stay, and earlier recovery.^1^ Despite advanced technology and improved surgical skills and knowledge, complication rates, including preventable injuries, are increasing. It is difficult to determine the exact incidence of complications. Definitions of complications vary and they are usually under-reported. The reported overall complication rates range from 0.2% to 10.3%. Major laparoscopic procedures are associated with a higher rate of complications.

Bowel injury is a serious complication of gynecological laparoscopy. Its incidence depends on the treated pathology and the type of procedure (diagnostic, minor operative, or complex operative). Lack of surgeon's experience and presence of previous abdominal surgery increase the risk of bowel injury. The incidence of bowel injury is 0.13% for laparoscopy procedures. The most common site of bowel injury was the small bowel, followed by the large bowel and stomach.^2^ This is in agreement with a recent systematic review which has shown that the incidence of bowel injury in gynecologic laparoscopy is 1 in 769.^3^

Published papers on this topic are mainly retrospective having heterogeneous populations, different diagnostic problems, different levels of operative laparoscopy skills, and different definitions of bowel injury.^4^ The indications for laparoscopy have changed over time with increased complexity defined by increased laparoscopic skills amongst gynecologists.

Bowel injury may occur during insertion of a Veress needle and trocar, use of electrosurgery and laser beams, suturing, and adhesolysis. Technical aspects play a major role in reducing these injuries. Majority of complications occur during entry into the abdomen and mainly during primary trocar entry.^5^ This made some surgeons advocate the use of open-entry technique. Nevertheless, this technique did not reduce bowel injuries.^6^ Majority of gynecologists use the Veress needle to create pneumoperitoneum.^7^ The incidence of injuries caused by Veress needle is reported to be 0.23%, out of which only 2.8% were bowel injuries.^8^ In this manuscript, we aimed to review prevention, diagnosis, and management of bowel injury following gynecological laparoscopic surgery. This was combined with personal experiences, so as to give recommendations on how to avoid and manage this difficult problem.

## Prevention

### Before surgery

Prevention of these bowel injuries should be started before surgery. The best prevention strategy includes careful analysis of the surgical history of the patient, the degree of complexity of planned surgery, the patient selection, and identifying surgical limits. Surgery on obese or thin patients, previous abdominal and pelvic surgery (in particular having midline scar), severe endometriosis, and complicated pelvic pathology should increase the surgeon's awareness of potential complications during laparoscopic procedures. Pre-operative bowel preparation for complicated surgery has been used by many gynecologists. This has potential benefits including reduction in infection and reduced anastomotic leakage following repair.^9^ Nevertheless, others have shown no benefit of bowel preparation in reducing complications.^10^

### During surgery

#### Veress needle insertion

Veress needle is commonly used by gynecologists to create a pneumo-peritoneum. Therefore, various safety tests have been done to determine its correct position.^11^ Teoh et al.^12^ have shown in a prospective study that Palmer saline test and double click test did not define the correct placement of the Veress needle. Traditionally, Veress needles are inserted at the base of the umbilicus because this area is very thin. The Palmer point entry for the Veress needle is 2–3 cm below the left sub-costal margin. This was recommended as an entry point for patients having lower midline scars, previous multiple abdominal surgeries, or intra-peritoneal adhesions.^13^ However, previous splenectomy and splenomegaly might be a contraindication for Palmer point entry. In addition, when Palmer point entry is attempted, it is recommended to deflate the stomach by placing nasogastric tube to avoid stomach injury.

#### Trocar insertion

**Figure 1:**
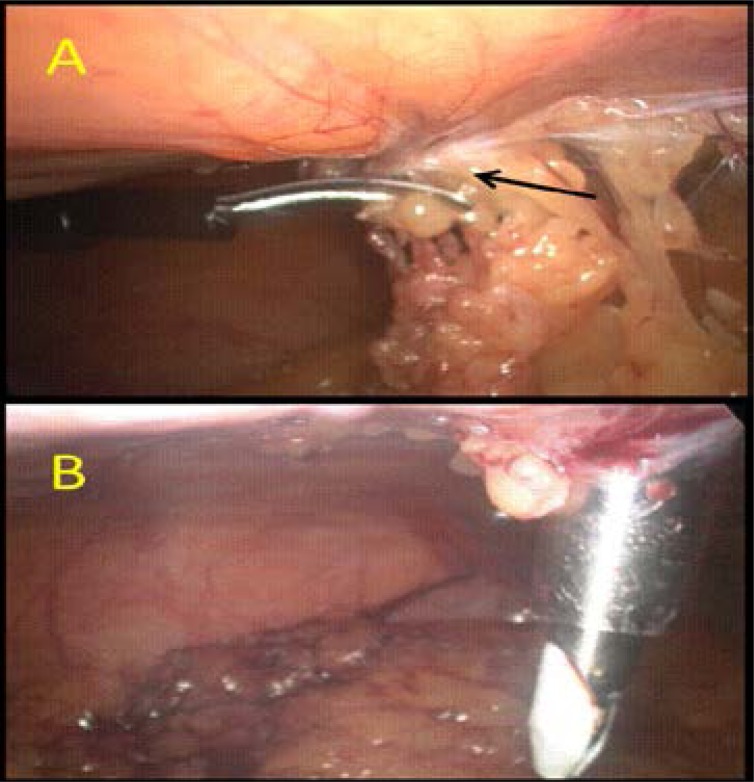
Identification and dividing of omental and bowel adhesion due to previous surgery is important prior to insertion of lateral ports: **A**: (black arrow = omental and bowel adhesion due to previous appendectomy), **B**: lateral port insertion under direct vision after releasing the omental and bowel adhesions.(Courtesy of Dr Hassan M Elbiss, Consultant Obstetrician and Gynecologist, Department of Obstetrics and Gynecology, College of Medicine and Health sciences, United Arab Emirates university, Al-Ain, United Arab Emirates).

Trendelenburg position should be avoided during primary trocar insertion because it can make the angle of insertion more perpendicular. In an average patient, the distance between the anterior abdominal wall and retroperitoneal vessels is normally 3 to 4 cm. It can be increased to 5.6 cm (range 4–8 cm) by creating a pneumo-peritoneum under pressure of 20–25 mmHg. The Royal College of Obstetricians and Gynaecologists^14^ recommend achieving a pressure of 20–25 mmHg before inserting the trocar. This increases splinting and allows the trocar to be more easily inserted through the abdominal wall. Following that, the procedure can be continued with an intra-abdominal pressure of 15 mm Hg. Careful inspection for omental and bowel adhesions prior to insertion of other trocars is important (Figure 1A).

Trocar screwing motion rather than direct pushing gives better control. The secondary trocars should be placed under direct vision to avoid vascular and internal organ injuries (Figure 1B). Using optical trocars did not reduce bowel injury.^15^

## New technology

In the last thirty years, several entry approaches, new instruments, and new techniques have been introduced to minimize laparoscopic complications. Electrosurgery during laparoscopy can be used for coagulation, dissection, cutting, and ablation. Electrosurgery-induced injury can be either direct mechanical or indirect electrothermal injury. The use of ultrasonic energy through a harmonic scalpel might reduce the risk of collateral damage. The development of microprocessor-controlled generators with feedback from the electrode-tissue interface to determine the power output with autostop facility has made bipolar energy even safer. However, experience with this technology is still primitive. Therefore, understanding the principles of electrosurgery and practicing it in simulation is important before using it in laparoscopic surgery. Newer hemostatic technologies such as Ultrasonic Technology which does not have electrosurgical current generated can be used to decrease the incidence of complications.

Electro-thermal injury can happen because of insulation failure, direct coupling, direct application and capacitive coupling. The incidence of electro-thermal injuries ranges between 2 to 5 per 1000 electrosurgical procedures and did not change over time.^16^ Therefore, gynecologists should understand the biophysics of electrosurgery, the function of its equipment, and its general tissue effects.

## Port closure

**Figure 2:**
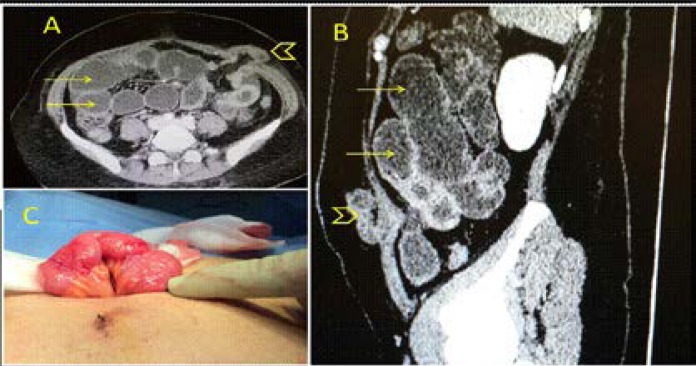
A 30-year-old female had a laparoscopic dermoid cystectomy. She developed abdominal pain, distension and vomiting 3 days after surgery. Abdominal CT scan with intravenous and oral contrast showed **(A–B)** distended small bowel loops (yellow arrows) and an incarcerated bowel loop in one of the ports (arrow head). The bowel was viable **(C)**, the port incision was extended, the bowel was reduced and the hernia was repaired (Courtesy of Dr Islam Sidky, Consultant Obstetrician and Gynecologist, Department of Obstetrics and Gynecology, Tawam Hospital, Al-Ain, United Arab Emirates).

Closure of a port of 10 mm or more has been recommended to avoid herniation of the small bowel^17^ (Figure 2). However, other factors including elderly patients, high body mass index, pre-existing hernia, the port size, trocar design, and increased time of surgery should be considered. 18

## Diagnosis

Early recognition of bowel injury and early intervention is crucial to reduce its morbidity and mortality.^19^ Despite care to identify bowel injury during surgery, only less than half of these injuries are diagnosed during laparoscopy. 5 When bowel injuries are not diagnosed during surgery, and dealt with at the same time, it may become a life-threatening condition. That is why it is the most common cause of laparoscopy-related death.^20^ A review of 66 cases showed that the mortality rate significantly increased if the diagnosis was delayed more than three days.^21^ The average time between surgery and diagnosis of small bowel injury was 3.3 days. However it was longer with an average of 4.8 days when injuries resulted from electrosurgery.^22^ The average time to diagnose large bowel injury was 1.3 days when sharp dissection was used and 10.4 days when electrosurgery was used. In a more recent study, 63% of missed bowel injuries were diagnosed two days or more after surgery.^23^

## On table recognition

**Figure 3:**
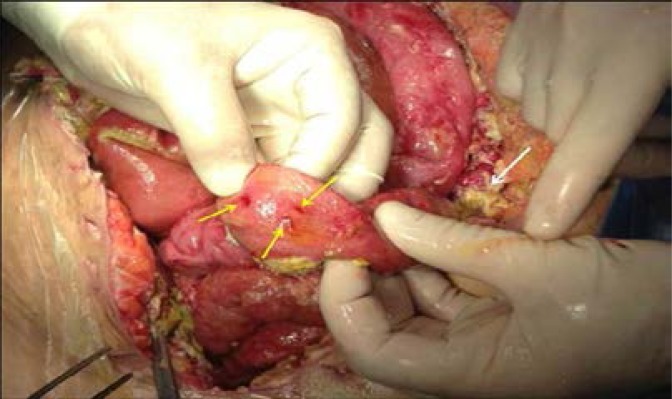
Multiple fine penetrations of a small bowel that were caused by the Veress needle having difficulty in insertion (yellow arrows). Peritonitis with purulent slough is seen at the background (white arrow) (Courtesy of Professor Farouk Safi, Consultant Surgeon, Saudi German Hospital, Dubai, United Arab Emirates).

Difficulty or repeat trials in creating adequate pneumoperitoneum should alert the surgeon to the possibility of bowel injury (Figure 3). This indicates careful inspection of the bowel surface to diagnose any bowel injury. If the bowel is entered by the Veress needle or trocar, then bowel contents or gas passage might be observed. Aspiration of bowel's content during Palmer saline test is highly suggestive of large bowel injury. Fecal odor might be noted if the large bowel was injured. Present of air bubbles when irrigating suspected injured bowel might be suggestive of bowel injury.^24^ The bowel should be always inspected following sharp or blunt dissection that in particular caused bleeding or hematoma so as to exclude bowel injury.

## Delayed diagnosis

A patient who does not improve after laparoscopy should be suspected to have unrecognized bowel injury. Delay in diagnosing a bowel perforation can lead to acute peritonitis and even death. If bowel injury is suspected, a general surgeon should be involved early in the management of these patients. The patient should be strictly observed and worked up to confirm or exclude the diagnosis. Work-up of these patients include serial abdominal examination and repeated laboratory and imaging studies as needed.

Symptoms from bowel injury generally manifest within twelve to thirty-six hours, but may occur up to five or seven days later. Presumably, patients who present after several days had either delayed necrosis of a damaged bowel, or had a leak which temporarily sealed off. Patients may develop non-specific complaints such as abdominal pain, intolerance of oral intake, bloating, nausea, fever or diarrhea. This may delay the diagnosis.^25^ Late presentations include generalized peritonitis, abscess formation, and septic shock. Repeated abdominal examinations, ideally conducted every 4–6 hours, are crucial for proper surgical decisions. A decision for laparotomy in diffuse peritonitis is mainly clinical. Nevertheless, abdominal examination in severe sepsis associated with altered mental status and those who are ventilated can be deceiving.^26^ Monitoring the white blood cell count may help in the diagnosis if significantly or persistently elevated. Procalcitonin and C-reactive protein should be correlated with the clinical findings to be useful. They cannot be used independently to make surgical decisions.^27^,^28^ Inflammatory markers are more useful, when their values are normal, to rule out infection. They are also very useful in the post-operative period in monitoring the patient's clinical progress.^29^

**Figure 4:**
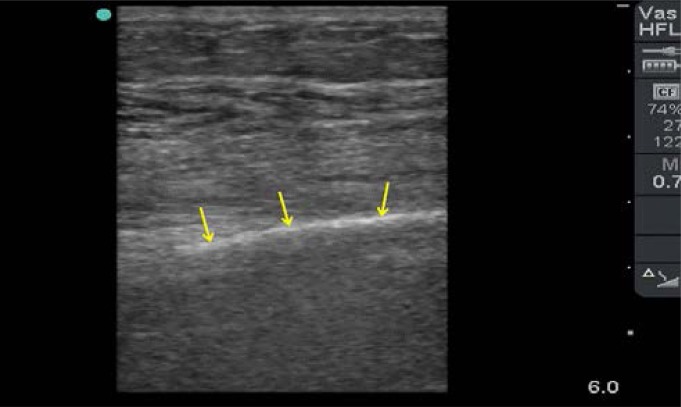
Ultrasound appearance of significant intra-peritoneal free air using a linear probe (10–12 MHZ). There is a hyper-echogenic line (yellow arrows) just under the abdominal fascia at the midline. It does not move with respiration but moves with repositioning the patient. It completely hides the organs deep to it (Courtesy of Professor Fikri Abu-Zidan, Department of Surgery, College of Medicine and Health Sciences, UAE University, United Arab Emirates).

The diagnosis can be established post-operatively if free intra-abdominal air on chest X-ray is increasing in size especially after 48 hours of surgery. Point-of-Care Ultrasound (POCUS) is a very useful portable diagnostic tool that can be repeated at bedside without risk of radiation. It is accurate in diagnosing both intra-peritoneal fluid and air.^30^ We have recently described in detail the ultrasound characteristics of intra-peritoneal free air.^31^ This appears as increased echogenicity of a peritoneal stripe accompanied by posterior reverberation parallel echogenic lines having equal distances that will hide the organs (Figure 4). Furthermore, ultrasound can be used to evaluate the function of the heart and the size of the IVC diameter which is useful in evaluating the hemodynamic status of septic patients.^32^ Nevertheless, it is operator dependable and less accurate than CT scan.

CT scan is more sensitive and specific than ultrasound. It should be used when an ultrasound study was not conclusive and a clinical suspicion of a significant intra-abdominal infection was present. The results of CT scan may support the clinical and ultrasound findings. CT scan has certain side effects including the risk of radiation, allergic reactions to contrast material, and renal toxicity.

## Surgical management

If recognized early and on table by an experienced laparoscopic surgeon, majority of bowel injuries can be repaired by laparoscopy or by mini-laparotomy.^19^,^24^ Nevertheless, early involvement of an experienced general surgeon is recommended whenever an intestinal injury is suspected during or following laparoscopic gynecological surgery. Laparoscopic repair of injury depends on its size and nature, and on the surgeon's experience.^33^

Injury caused by the Veress needle can be managed conservatively since its small diameter leaves no defect and the muscular layer will close the defect. Trocar injuries may require a laparotomy for repair. When trocar injury occurs, leaving the trocar in place may be useful to identify the site of injury.^34^

The extent of bowel injury caused by bipolar electrosurgery can be easily identified. Nevertheless, it is difficult to evaluate and identify those resulting from monopolar electrosurgery.^16^ Therefore, excision of the damaged area and up to 5 cm around the margin of injury is recommended to prevent subsequent perforation.^35^ Conversion to laparotomy should be considered when laparoscopic management is not accessible or safe, and in cases of delayed diagnosis of bowel injury so as to evaluate the entire abdominal cavity.

Primary closure in two layers using 3/0 Vicryl or PDS sutures is usually sufficient for majority of small bowel injuries. In case of a large bowel injury, the treatment options include primary repair, segmental resection, or colostomy. Segmental resection is more common in thermal injuries. There is strong evidence supporting primary repair of the colon and avoiding a colostomy especially in hemodynamically stable patients.^36^

**Figure 5:**
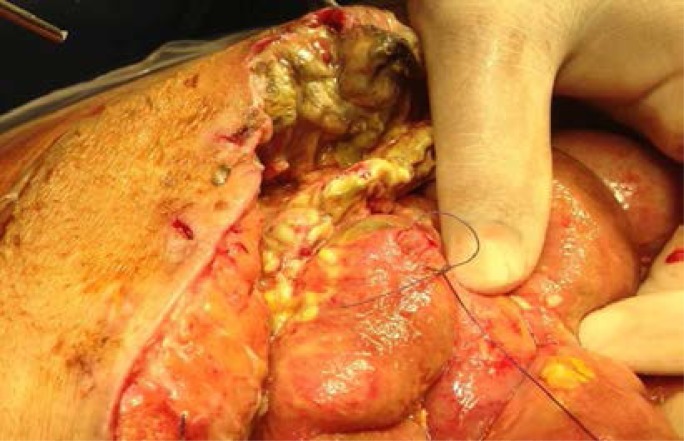
A 5 mm laparoscopic small bowel injury that was treated with primary closure (Courtesy of Professor Farouk Safi, Consultant Surgeon Saudi German Hospital, Dubai, United Arab Emirates).

When bowel injury is less than 2 cm, the bowel might be repaired transversely or longitudinally, although transverse closure to reduce the risk of stenosis is recommended (Figure 5). However, when the injury is more than 2 cm, it should be repaired transversely. A colonic injury with minor contamination can have primary repair. When laceration is more than one-half of the diameter of the lumen, or when mesenteric blood supply is involved (regardless of the length of laceration), segmental resection and anastomosis is indicated.

In severe cases of delayed peritonitis having a risk of abdominal compartment syndrome, the abdomen can be temporarily left open without closing the abdominal fascia. The fascia can be closed later on when the source of infection is controlled.^37^

## Future perspectives

Ideally, the best and cheapest solution for bowel injury is prevention. Bowel adhesion is a potential risk factor for bowel injury. Different anti-adhesive agents have been developed and tested to reduce adhesion formation. Nevertheless, strong evidence to support their general use is still lacking.^38^

Robotic surgery allows less experienced laparoscopic surgeons to perform more complex gynecological procedures because it improves visualization and access. Whether it reduces bowel injuries needs to be further investigated. 39 Three-Dimensional (3D) laparoscopic gynecological surgery was developed to provide the surgeon with a monitor image that closely resembles actual anatomy. This improves the speed and accuracy of laparoscopic phantom tasks and offers an advantage in teaching laparoscopic skills. Yet, the impact of 3D laparoscopic gynecological surgery on bowel injury needs to be assessed. 40

In summary, bowel injury remains a potential serious complication of gynecological laparoscopy. Every effort should be made to prevent it. Early recognition of bowel injury and its management is crucial for a favorable clinical outcome. Work-up of patients who are suspected to have missed bowel injury include serial abdominal examination, measuring inflammatory markers, and performing imaging studies as needed. A combined collaboration between laparoscopic gynecologists and general surgeons is important for timely proper decisions to be made in these difficult cases.
